# Xenotropic murine leukemia virus-related virus (XMRV) in prostate cancer cells likely represents a laboratory artifact

**DOI:** 10.18632/oncotarget.287

**Published:** 2011-06-02

**Authors:** Jiawen Yang, Partho Battacharya, Ruchi Singhal, Eugene S. Kandel

**Affiliations:** ^1^ Roswell Park Cancre Institute, Department of Cell Stress Biology, Elm & Carlton St., Buffalo, NY, 142263, USA; ^2^ City Honors School, 186 East North Street, Buffalo, NY, 14204, USA

**Keywords:** XMRV, prostate cancer, CWR22, 22Rv1

## Abstract

The prevalence of xenotropic murine leukemia virus-related virus (XMRV) in human population and its involvement in prostate cancer are subjects of ongoing research and debate. 22Rv1, which is a human cell line that serves as a common model of androgen-independent prostate cancer, was recently reported to carry infectious copies of XMRV. 22Rv1 was derived from a prostate cancer xenograft CWR22 that was serially passaged in immunodeficient mice. Based on the analysis of the DNA from CWR22 and 22Rv1, we present evidence against the presence of XMRV in CWR22 and, by inference, the tumor, from which CWR22 and 22Rv1 were established. While the presence of XMRV in 22Rv1 is likely to be an artifact, it may be a significant factor in determining the biological properties of this cell line. This consideration warrants additional caution for the interpretation of the relevance of the studies, which utilize this popular cell line as a model. It also invites a closer look at the sources of viral contamination in xenografts and cultured cells, as well as in the experiments that allege the presence of this virus in human cells and populations.

## BACKGROUND

Xenotropic murine leukemia virus-related virus (XMRV) is a recently discovered human gammaretrovirus that shares a very high degree of homology with murine leukemia virus (MLV)[1, 2]. XMRV was first identified in samples from prostate cancer patients and was reported to be more prevalent in the individuals with mutations in RNAse L gene. Subsequent studies reported very high incidence of XMRV infection among the individuals diagnosed with chronic fatigue syndrome. The same studies reported noticeable presence of XMRV among apparently asymptomatic individuals, suggesting that several percent of the studied control populations may be carriers of the virus. Numerous subsequent studies significantly differ in their conclusions on the incidence of XMRV infection in healthy individuals, as well as on its association, if any, with chronic fatigue, prostate cancer or RNAse L mutations [3-12].

Originally, XMRV was detected in fibroblast and, more rarely, hematopoietic cells of prostate cancer patients. This is in a sharp contrast with rodent tumors caused by murine leukemia virus, where the virus is ubiquitously found in the tumor cells. This prompted discussions about the role, if any, that the virus might have in the etiology of the disease. Later studies did report detection of XMRV in cancer epithelial component [13], although the causative role of the virus remained open to discussion. In this regard, a potentially important finding was made when XMRV was detected in a human carcinoma cell line 22Rv1[14]. Unlike patient-derived samples, this cell lines provides a virtually unlimited supply of material for investigation, which greatly extends simplicity and reliability of analysis. In addition to yielding a concrete example of XMRV presence in prostate cancer cells, these findings potentially provided an avenue to test the causal link between the virus and the transformed phenotype of the cells. For example, the proviruses may be tested for the presence of recombinant oncogenes, and the genes adjacent to the insertion sites and, possibly, affected by the provirus may be tested for their involvement in oncogenesis.

An important pitfall en route to linking the XMRV infection and the transformed status of 22Rv1 is that the presence of the virus in the cultured cells does not prove that the virus was present in the original tumor. The cell line was established from a human xenograft that was first serially-passaged in immunocompromised mice and then was extensively cultured in vitro. Therefore, there is a possibility that the virus was introduced either in the mouse host or during culture. The risk of the later is underscored by the propensity of XMRV to spread between cultured human cell lines [2, 15].

Although the original tumor specimen is unavailable to us, we decided to investigate whether XMRV is present in an early-passage CWR22 xenograft.

## RESULTS

We have obtained a fragment of an early passage CWR22 xenograft from the Roswell Park Cancer Institute collection, and the 22Rv1 cell line from the laboratory of Dr. Gudkov at the same institution. Because misidentification of the origin of cultured cells is a common problem, we used the analysis of polymorphic sites to confirm that the cell line and the xenograft are singeneic. Indeed, every single one of the 65 tested polymorphic sites was identical between the two DNA samples.

**Figure 1:**
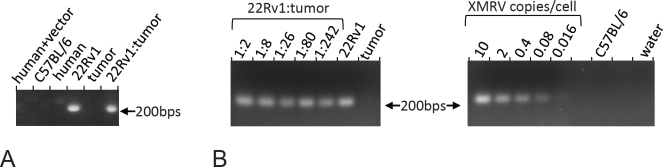
PCR amplification of XMRV sequences from genomic DNA and model mixtures A. PCR with XMRV-specific primers was performed on the DNA from human cells infected with an MLV-based vector (“human+vector”), a C57BL/6 mouse (“C57BL/6”), uninfected human prostate cancer cells (“human”), 22Rv1 cells (“22Rv1”), CWR22 tumor, and on a 1:2 mix of 22Rv1 and CWR22 DNA (“22Rv1:tumor”). B. The indicated model mixtures were created using the DNA from 22Rv1 cell line and CWR22 tumor, as well as by spiking CWR22 tumor DNA with appropriately diluted plasmid, which contained a complete XMRV genome. PCR was also performed on pure DNA from 22Rv1, CWR22 (“tumor”), and a C57BL/6 mouse, as well as on the water control. Some wells were deliberately left blank.

Next, we designed a PCR strategy that would reliably and specifically identify the presence of XMRV DNA in genomic samples. The main challenge in this undertaking is avoiding cross-reactivity with the endogenous retrovirus in human and, especially, mouse genome [16-19], as well as with the commonly used retroviral vectors. To this end, we took advantage of a characteristic deletion, which sets XMRV aside from most endogenous mouse viruses. We were able to achieve a robust and specific amplification of control XMRV DNA. As is seen on a representative gel (Fig. 1A), no product was generated from mouse DNA, human DNA from prostate cancer cells, as well as from human DNA from a cell line that harbors an integrated retroviral vector. As expected, 22Rv1 cells produced a strong band. The fragment amplified from 22RV1 cells was cloned and sequenced and revealed 100% homology with the sequence of XMRV previously identified in these cells[14]. The attempts to amplify the fragment from the DNA of the CWR22 tumor failed. This is not due to the presence of an inhibitor in the tumor DNA: detectable signals were obtained from the tumor DNA reconstituted with various quantities of XMRV DNA in a plasmid form, as well as with the DNA from the 22Rv1 cells (Fig. 1B). The absence of the signal in some of the samples, the water controls and in the extreme dilutions of the positive controls with the tumor DNA confirms the specificity and indicates the limits of sensitivity of our procedure.

**Figure 2:**
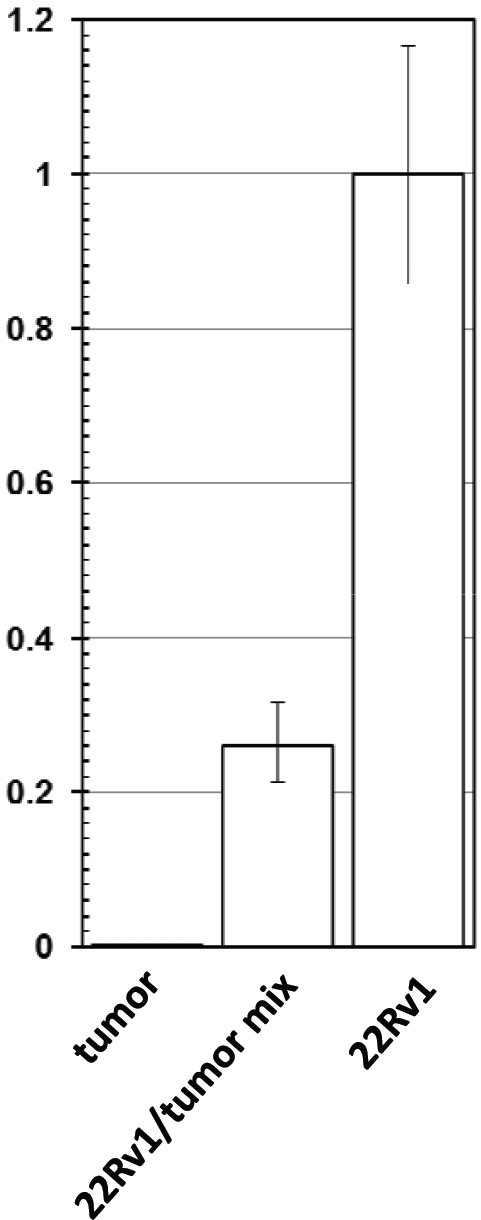
Detection of XMRV genome by quantitative PCR Quantitative PCR using XMRV-specific primers was performed on pure DNA from 22Rv1 (“22Rv1”) and from CWR22 xenograft (“tumor”), as well as on a 1:3 mixture of the two (“22Rv1/tumor mix”). Amplification of a fragment of human myogenin promoter was used as an internal control. The data was normalized for the value in 22Rv1, and is shown as an average with standard deviation.

To account for the possible differences in the quality of the DNA from 22Rv1 and the CWR22 tumor we have conducted quantitative real-time PCR, using a pair of primers for a fragment of human myogenin promoter as an internal control. While the efficiency of amplification of the internal control was similar in all samples, 22Rv1 yielded a robust signal, the signal from the DNA of the CWR22 tumor was negligible (at least 370 fold lower than in 22Rv1 sample), and the mixture of the two showed the expected intermediate result (Fig. 2).

The sensitivity of our approach cannot rule out that some individual cells in the CWR22 tumor carry XMRV. However, based on our model dilutions and the prior reports that XMRV load in 22Rv1[14], we concluded that the virus load in the xenograft is significantly lower that one copy per cell.

## DISCUSSION

Our results indicate that XMRV virus has not spread in CWR22 tumor before it was removed from the patient and serially passaged as a xenograft. The mechanisms of oncogenesis previously associated with infection with MLV-like retroviruses include insertional mutagenesis and transduction with oncogenes serendipitously incorporated into the viral genomes. Both of these mechanisms imply the presence of at least one particular copy of the provirus in every cancer cell and, therefore, are ruled out by our observations of CWR22 xenograft. Although we cannot technically rule out that a very small fraction of cells in the examined CWR22 xenograft did carry the virus, we find it unlikely, that a replication-competent virus would fail to spread in a pool of susceptible cells after multiple passages in an immuno-deficient host. Therefore, we conclude that, in the most likely scenario, the virus was not present in the tumor in the original patient, and has been introduced as an artifact later. This conclusion raises important questions about the sources of this contamination, and invites scrutiny to any other findings of the virus in cultured cells. It is important to note that our results do not rule out the presence of XMRV in human population in general, but bring about the questions about the sources of contamination that might have influenced the interpretation of results from patient samples.

While this paper was in preparation, another report [19] has suggested that XMRV presence in human patient samples is an artifact of the PCR techniques used to detect the virus. Although the authors of that report did not provide the evidence that their own detection technique was free of contamination, they mention that XMRV-like sequences were found in some of the tested inbred mouse strains (apparently, not examined were the immunocompromized strains that are typically used to harbor human xenografts), and suggest an intriguing hypothesis that the XMRV sequences allegedly derived from the patients emerged through recombination between XMRV from 22Rv1 and some endogenous murine viruses. In this regard, it is important to note that the mouse DNA used in the current study was derived from a strain, which was extensively back-crossed to C57BL/6J background. The recent report failed to identify the characteristic XMRV sequences in C57BL/6J DNA[19], and the Primer-BLAST analysis (accessed through http://www.ncbi.nlm.nih.gov/tools/primer-blast/) indicated that the primer pair used in our study lacks targets in the mouse genome.

22Rv1 is a widely-used in vitro model of prostate cancer. It has been especially popular as a model of the androgen-independent form of the disease. It has been a subject of numerous studies that try to identify the determinants of the resistance, the ways to overcome it, as well as additional potential targets for the therapy of this malignancy. Establishment of prostate cancer cell lines that retain meaningful properties of the disease is notoriously difficult. In this regard, it is important to mention that the original tumor and the routinely passed CWR22 xenografts were androgen-dependent, while the cell line was established from a xenograft that recurred after castration of the host [20]. The material from the specific xenograft immediately prior to the selection for androgen-independence, as well as from the earliest passages of the cell line, is not available to us. At this time, one cannot rule out the possibility that XMRV infection played a role either in establishment of androgen-independence or in adaptation of the cells to culture conditions. Prior analyses of proviruses in the 22Rv1 DNA are consistent with the presence of multiple copies of unrearranged full-length virus in every cell. Oncogenic effects of unrearranged MLV are attributable to insertional mutagenesis[21]. In this case, insertion of the LTR-encoded promoter underlies the high yield of phenotypically-detectable mutations [22]. Consequently, the function of the promoter becomes a critical determinant of the phenotype of the cell. XMRV integration site preferences make it a plausible mutagen[23], while the initial analysis of the functional organization of the XMRV promoter revealed important differences from the classical MLV, including functional sites of hormone-regulated transcription factors[24]. If the function of the LTR promoter indeed contributes to the properties of this cell line, then one may discover various dependencies and vulnerabilities in 22Rv1, which are related to the regulation of the LTR, but are irrelevant to the properties of “real-life” human tumors. We believe that before the role of XMRV in the biology of 22Rv1 has been clarified, the implications of the studies conducted on this cell line should be treated with great caution.

In conclusion, the lack of detectable presence of XMRV in CWR22 xenograft argues that the virus was also absent in the original tumor, and its presence in 22Rv1 is an artifact. While the involvement of XMRV in determining biological properties of 22Rv1 remains unknown, the clinical relevance of the findings in this model of prostate cancer warrants extra scrutiny.

## METHODS

### Polymerase chain reaction

XMRV DNA was amplified using XMRV2AS (GTCCCCCAACAAAGCCACTC) and XMRV2S (ATCTAATCCTCGCGCCTGCGTC) primers. Myogenin-Forward (GTTTCTGTGGCGTTGGCTAT) and Myogenin-Reverse (GGTCGGAAAGGGCTTGTT) primers were used to amplify a fragment of human myogenin promoter. End-point PCR was done using Cheetah Taq (Biotium) for 36 cycles (94° C for 30 s, 56° C for 30 s, 72° C for 45 s). For quantitative PCR, iQ SYBR Green Supermix (Bio-Rad) was used. The samples were cycled (94°C for 30 s, 56°C for 30 s, 72°C for 45 s) in My iQ Single Color Real-Time PCR Detection System (Bio-Rad); and the results were processed using manufacturer's software and the ddCt method.

### Single nucleotide polymorphism genotyping

SNP genotyping was performed using the MassARRAY Compact system (Sequenom) on a panel of 65 custom SNP assays designed using RealSNP and MassARRAY Assay Designer (Sequenom). Briefly, the protocol involves PCR amplification of 10ng DNA using SNP specific primers, followed by a base extension reaction using the iPLEX Gold chemistry (Sequenom). The final base extension products were treated and spotted on a 384-pad SpectroCHIP (Sequenom) using a ChipSpotter LT nanodispenser (Samsung). A MassARRAY Analyzer Compact MALDI-TOF MS (Sequenom) was used for data acquisition from the SpectroCHIP. The resultant genotypes were called using MassARRAY Typer Analyzer v4.0 (Sequenom).
